# Champaign County Medical Society

**Published:** 1868-05-01

**Authors:** Samuel H. Birney

**Affiliations:** Secretary


### CHICAGO MEDICAL SOCIETY.

### CHAMPAIGN COUNTY MEDICAL SOCIETY.

The fourth regular session of the society was held at the office of Dr. C. H. Mills, in Champaign City, Wednesday, the 1st of April, 1868.

The association was called to order by the president in the chair. Minutes of the previous meeting were read and approved.

A very interesting paper was read on the use of the speculum in the treatment of uterine diseases, which was discussed with spirit and profit.

The following officers have been elected for the ensuing year.

*President*—Dr. C. H. Mills; *Secretary*—Dr. S. H. Birney; *Senior Vice-President*—Dr. W. Somers; *Junior Vice-President*—Dr. W. R. Earheart; *Treasurer*—Dr. I. T. Pearman; *Censors*—Dr. H. C. Howard, I. McHugh, M. S. Brown. Delegates to Illinois State Medical Association—Dr. W. Earheart, and Dr. S. H. Birney.

On motion, the secretary was directed to prepare an account of the association for publication, and send to the Chicago Medical Journal.

On motion, the society adjourned to meet in Urbana the first Wednesday in June.

I am happy to state that the society is in a very flourishing condition.

SAMUEL H. BIRNEY, *Secretary*.

